# Gyrovirus: current status and challenge

**DOI:** 10.3389/fmicb.2024.1449814

**Published:** 2024-08-16

**Authors:** Tianxing Yan, Zhuoyuan Wang, Ruiqi Li, Dabin Zhang, Yuchen Song, Ziqiang Cheng

**Affiliations:** Department of Basic Veterinary Medicine, College of Veterinary Medicine, Shandong Agricultural University, Tai’an, China

**Keywords:** gyrovirus, epidemiology, genetic evolution, pathogenicity, immunopathogenesis

## Abstract

Gyrovirus (GyV) is small, single-stranded circular DNA viruses that has recently been assigned to the family *Anelloviridae*. In the last decade, many GyVs that have an apparent pan-tropism at the host level were identified by high-throughput sequencing (HTS) technology. As of now, they have achieved global distribution. Several species of GyVs have been demonstrated to be pathogenic to poultry, particularly chicken anemia virus (CAV), causing significant economic losses to the global poultry industry. Although GyVs are highly prevalent in various birds worldwide, their direct involvement in the etiology of specific diseases and the reasons for their ubiquity and host diversity are not fully understood. This review summarizes current knowledge about GyVs, with a major emphasis on their morphofunctional properties, epidemiological characteristics, genetic evolution, pathogenicity, and immunopathogenesis. Additionally, the association between GyVs and various diseases, as well as its potential impact on the poultry industry, have been discussed. Future prevention and control strategies have also been explored. These insights underscore the importance of conducting research to establish a virus culture system, optimize surveillance, and develop vaccines for GyVs.

## 1 Introduction

The Anelloviruses family includes numerous circular single-stranded (ss) DNA viruses that includes many viruses that infect a wide range of animal species, including humans. The family includes 31 well-established genera (Varsani et al., 2021), the genus *Gyrovirus* (GyV) known to infect various avian species, while the remaining 30 genera primarily infect humans and various mammal species (Spezia et al., 2023). Most of the research regarding anelloviruses has focused on torque teno virus (TTV), which is closely linked to humans, while the impact of anelloviruses on economically beneficial animals has been neglected. Chicken anemia virus (CAV), the first member of the genus GyV, has been revealed to be pathogenic to chickens and has caused significant economic losses to the poultry industry worldwide (Yuasa et al., 1979; Schat, 2009). Apart from the widely recognized CAV, multiple newly GyVs have been found in most environments and in association with diverse hosts. Although these GyVs have been identified in human skin, human feces, chicken serum, meat, and various tissues from wild birds (Rijsewijk et al., 2011; Chu et al., 2012; Maggi et al., 2012; Phan et al., 2012; Biagini et al., 2013; Gia Phan et al., 2013; Fehér et al., 2014; Zhang et al., 2014; Li et al., 2015; Phan et al., 2015; Goldberg et al., 2018; Waits et al., 2018; Truchado et al., 2019; Cibulski et al., 2020; Loiko et al., 2020; Fehér et al., 2022; Wierenga et al., 2023), their pathogenicity remains largely unexplored due to the scarcity of virus isolates and the lack of a virus culture system. They may act as cofactors in the onset and progression of disease in chicken, such as transmission viral proventriculitis (TVP). Recent studies have revealed the role of GyV in chicken-related diseases, which has led to new hypotheses regarding the potential pathological mechanisms of these viruses (Li et al., 2021; Yuan et al., 2021; Zhang S. et al., 2022). This review, summarizes the relevant literature on GyVs studies, aiming to improve our understanding of viral origin, transmission, host, pathogenicity, and immunopathogenesis, and assist in developing assays, classification and characterization in GyV.

## 2 Genome structure and function

**Figure 1:**
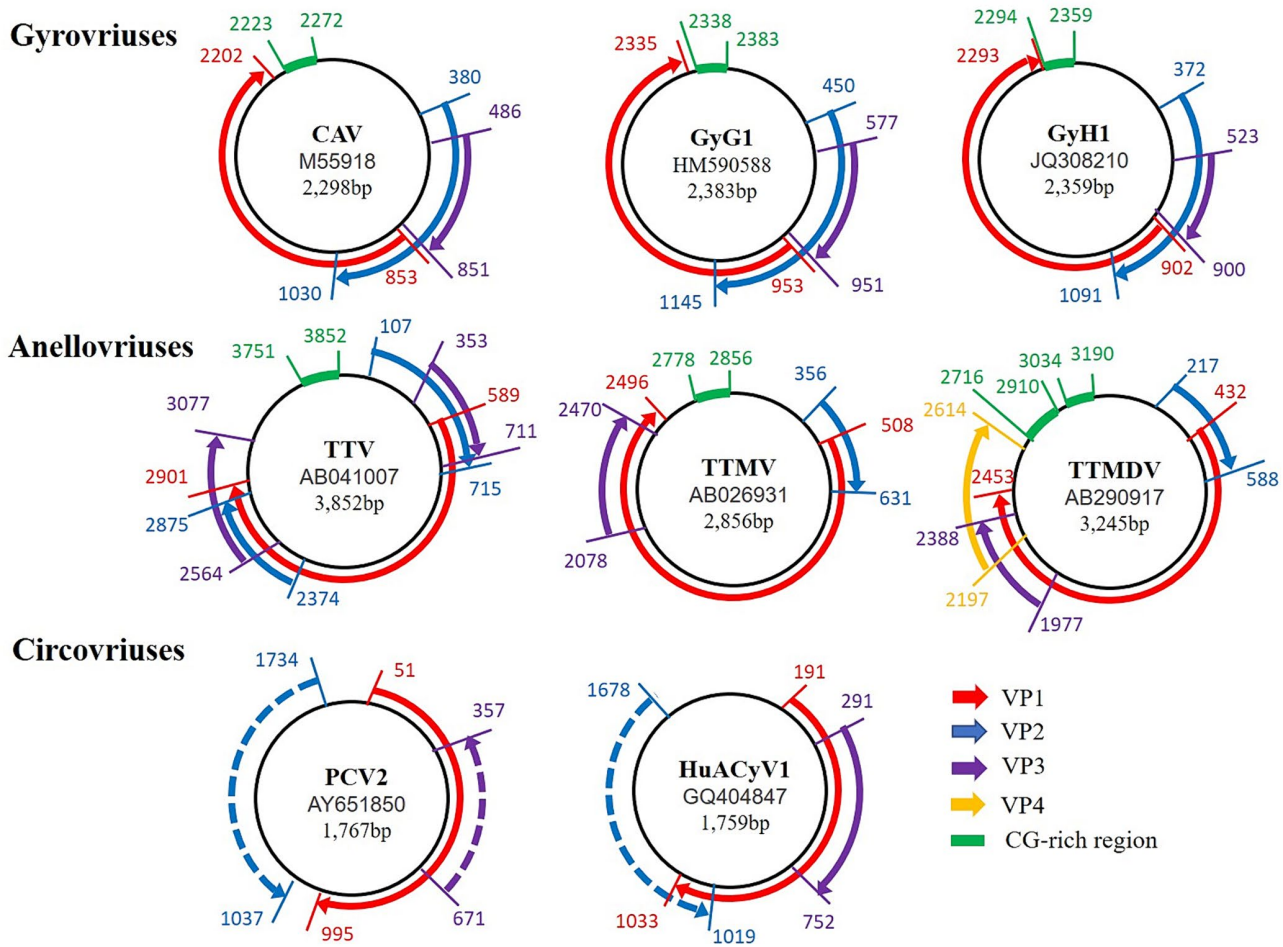
Comparison of the genomic organization of Gyrovriuses, Anellovriuses, and Circovriuses. The access number and the genome size for each of the isolates appear in the center of its corresponding circular negative-sense single-stranded DNA genome (represented in each case by the innermost black circle). The direction, relative size, and reading frame of each ORF are indicated by the location, length, and color, respectively, of the arrows located externally to the circular genomic DNA.

The genetic structure of GyV is a single-stranded circular negative-sense DNA of 1.8–2.4 kb in size, containing three overlapping ORFs and an untranslated region (UTR) (Noteborn et al., 1994). These proteins are translated from a single multicistronic mRNA by start codon alternation. The mechanism of codon alternation has not yet been elucidated. The current identified GyVs share the synteny of their genomes (Figure 1).

ORF1 encodes the viral scaffolding protein VP2. The VP2 of CAV has dual-specificity phosphatase (DSP) activity, which catalyzes the dephosphorylation of phosphoserine, phosphothreonine, and phosphotyrosine substrates. This activity is involved in viral replication, cytopathology, and pathogenesis (Peters et al., 2006). The active motifs CX_5_R and WX_7_HX_3_CXCX_5_H are also widely presented in the VP2 of other GyVs.

ORF2 encodes the viral protein VP3, also known as apoptin (no ORF encoding the apoptotic protein VP3 was found in GyH3, GyM1, GyPi1, and GyA2). This protein induces apoptosis in chicken T lymphocytes and hematopoietic cells, causing anemia in infected chickens, and selectively triggers apoptosis in tumor cells (Noteborn, 2004). Apoptin contains both a putative nuclear export as well as a putative nuclear localization signal. The VP3 of GyG1, GyH1, and GyM1 have the same conserved sequences of nuclear localization signals and nuclear output signals as CAV (Rijsewijk et al., 2011; Phan et al., 2012; Truchado et al., 2019). The tumor-cell killing ability of apoptin has generated significant interest for its potential as an anticancer therapy, and indeed apoptin has been investigated extensively in a wide range of human tumor cells, both *in vitro* and *in vivo*, including melanoma, hepatoma, osteosarcoma, lung carcinoma, breast cancer, and prostate cancer (Backendorf et al., 2008; Los et al., 2009; Feng et al., 2020).

**Figure 2:**
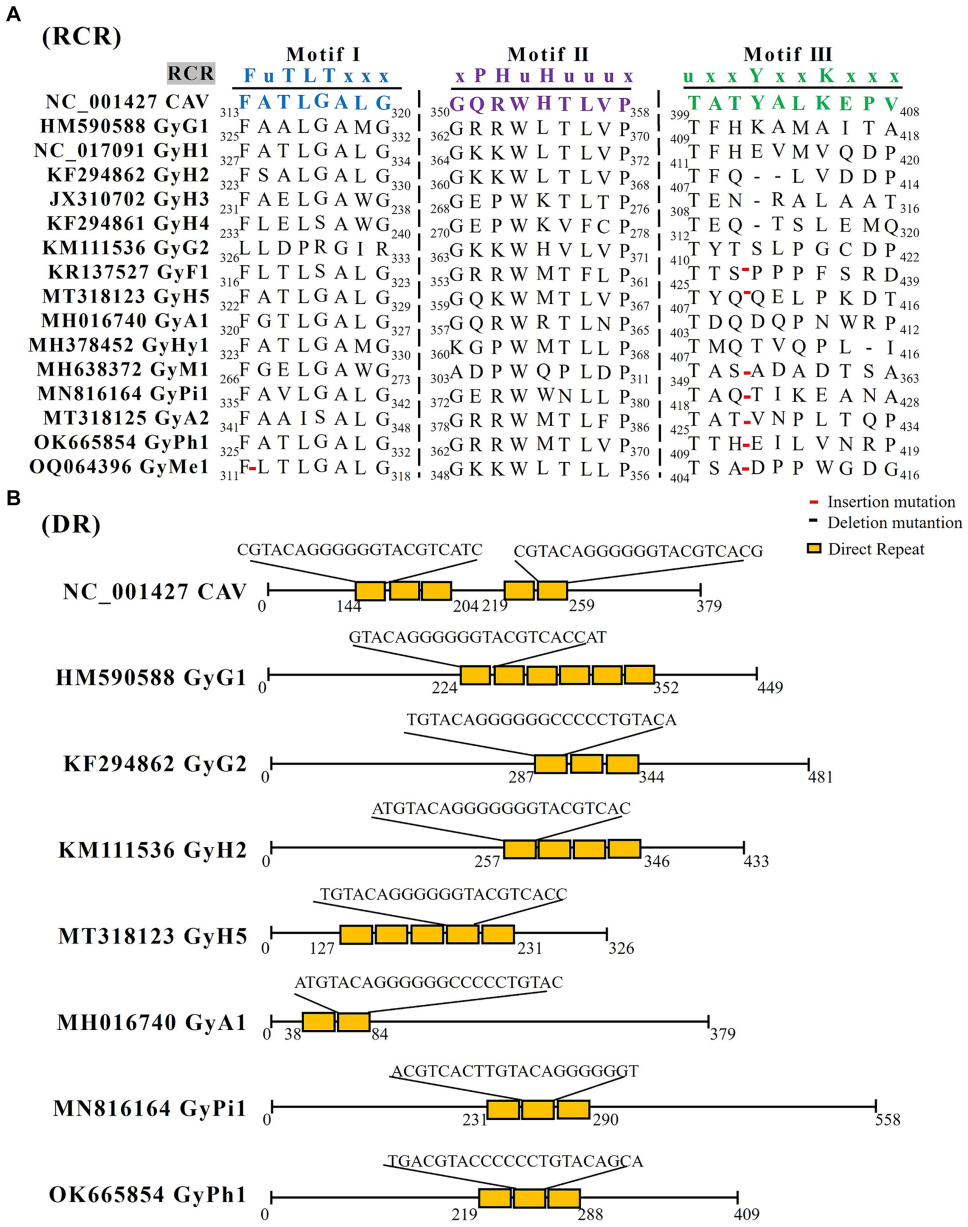
The RCR and DR analysis of GyV. **(A)** GyV VP1 amino acid sequence alignment using Clustal W in MEGA 6 for screening RCR motif. Red lines represent insertion mutations, black lines represent deletion mutations. **(B)** The DR analysis of the non-coding regions of GyV was performed using Tandem Repeats Finder (https://tandem.bu.edu/trf/home) (Benson, 1999). The yellow squares represent the DR sequences for each GyV strain.

ORF3 encodes the viral capsid protein VP1, the only structural protein of the GyV. Although the capsid contains only VP1, co-expression of VP2 is required for the induction of neutralizing antibodies (Koch et al., 1995). The VP1 of CAV has a DNA binding function at its N-terminal end and three rolling circle replication (RCR) related peptide chain motifs at its C-terminal (Todd et al., 2001). Reps can be identified by the presence of three conserved motifs including motif I, motif II, and motif III (Ilyina and Koonin, 1992). Although CAV contains the conserved RCR motifs, these motifs are not conserved in other GyV, and only motif I of GyH1, GyH5, and GyPh1 is identical to that of CAV (Figure 2A). There are many insertion and deletion mutations in motif III. Importantly, these motifs are different from the family *Circoviridae*, which will help us to better identify GyVs.

The 5′ UTR was identified as the sole promoter/enhancer region that controls viral transcription and replication. The CAV UTR region contains five promoter direct repeat (DR) sequences. The DR contains a putative estrogen response element; estrogen activates and upregulates viral transcription (Noteborn et al., 1998; Miller et al., 2005). The number of DRs varies in other GyVs UTRs, with six in GyG1, three in GyG2, three in GyH2, five in GyH5, two in GyA1, three in GyPi1, and three in GyPh1. These DR sequences length between 21 bp to 23 bp (Figure 2B). Moreover, the UTR region immediately downstream of VP1 contains a section with high GC content, which complicates the sequencing and amplification processes of the entire viral gene. The UTR of GyH1 is more similar to that of CAV (62%) compared to GyG1 (37–47%) (Gia Phan et al., 2013; Li et al., 2015; Phan et al., 2015). The GC high-content region, which is highly similar (94% similarity of 109 bases), is shared between GyH1 and CAV, suggesting high conservation of the high-GC region that could be under negative selection rather than the result of a recombination event. Evidence for the putative recombination event is not presented (Phan et al., 2012).

The replication pattern of GyV remains unclear, however, the prevailing view is that its DNA is replicated through RCR (Todd et al., 1996). It is well established that the virus does not possess a mechanism to replicate its DNA and instead relies on host cells to do so, acquiring a double-stranded replicative form (dsRF) in the process (Claessens et al., 1991; Noteborn et al., 1991; Schat, 2009). Arguments supporting this view include the lack of a replicase-related (Rep) protein and sequences required for binding to dNTPs in VP1. Additionally, the conserved stem-loop structure containing the TAGTATTAC nucleotide sequence, which is associated with the initiation of DNA replication in RCR, is only partially conserved in CAV (Meehan et al., 1992).

## 3 Taxonomy and naming

CAV became the type species of the family *Circoviridae* when it was originally reported as an official virus family in 1995 with the release of 6th report by the International Committee on Taxonomy of Viruses (ICTV) (Murphy et al., 1995). It was presently realized that CAV did not share the same characteristics as beak and feather disease virus (BFDV) and porcine circovirus (PCV), accordingly, a second genus, GyV, was created within the family *Circoviridae* in 1999 to accommodate CAV (Todd et al., 1991). However, the accumulation of morphology, genomic structure, and molecular data indicated that GyV represents a distinct lineage of ssDNA viruses as originally suspected. GyV virions are non-enveloped and icosahedral; they are larger than circovirus virions and have a unique structure with protruding pentagonal shaped units compared with the flat pentameric units observed in circoviruses (Crowther et al., 2003). In addition, GyV has no genetic or structural similarity to members of the *Circoviridae* family (Figure 1). Instead, the genomic features of GyV are reminiscent of members of the *Anelloviridae* family (Bassami et al., 1998; Hino and Prasetyo, 2009; de Villiers et al., 2011). Based on this, the ICTV reclassified GyV as *Anellovirade* in 2016 (Rosario et al., 2017).

In 2021, Kraberger et al. proposed a species demarcation criterion of 69% based on the VP1 nucleotide sequence pairwise identity thresh old for species demarcation also for GyV (Kraberger et al., 2021). Based on this criterion, they established nine new species to accommodate the 49 unclassified GyVs and adopted the binomial nomenclature “Genus + freeform epithet” (Siddell et al., 2020). However, between then and now, six GyVs remain unclassified. We compared the VP1 nucleotide homology between these six GyVs and existing GyVs and showed less than 69%. According to the species demarcation criterion for GyV, we established an additional six species to accommodate these GyVs. Recently, Varsani et al. provided an update on the revised species names (using a freeform alphanumeric epithet wherever possible) for all species in the *Anelloviridae* family (Varsani et al., 2023). The genus GyV have previously been named with a binomial freeform alphanumeric epithet format, which consists of the five-letter epithet derived from the host species followed by a number. According to this naming standard, we named the six newly established GyVs (GyH5, GyA1, GyA2, GyPi1 GyPh1, and GyMe1). To date, there are a total of 16 species of GyVs, including CAV. The details are displayed in Table 1.

**Table 1:** Information about a member of the genus GyV.

Time	Country	Virus		Host	Source	Pathogenicity	References
		Old name	New name				
1979	Japan	Chicken anemia virus (CAV)	Gyrovirus chicken anemia	*Gallus gallus*	Vaccine	Anemia, immunosuppression	Yuasa et al. (1979)
2011	France	Human gyrovirus/Avian gyrovirus	Gyrovirus galga1 (GyG1)	*Gallus gallus*/*Homo sapiens*	Serum	Neural symptom	Rijsewijk et al. (2011), Maggi et al. (2012)
2012	Chile	Gyrovirus 3	Gyrovirus homsa1 (GyH1)	*Homo sapiens*	Feces	Anemia, TVP, immunosuppression	Phan et al. (2012)
2012	China	Gyrovirus 4	Gyrovirus homsa3 (GyH3)	*Homo sapiens*	Feces	Unknown	Chu et al. (2012)
2013	Tunisia	Gyrovirus 5	Gyrovirus homsa2 (GyH2)	*Homo sapiens*	Feces	Unknown	Gia Phan et al. (2013)
2013	Tunisia	Gyrovirus 6	Gyrovirus homsa4 (GyH4)	*Homo sapiens*	Feces	Unknown	Gia Phan et al. (2013)
2014	USA	Gyrovirus 7	Gyrovirus galga2 (GyG2)	*Gallus gallus*	Meat	Unknown	Zhang et al. (2014)
2015	USA	Gyrovirus 8	Gyrovirus fulgla1 (GyF1)	*Fulmarus glacialis*	Spleen	Neural symptom	Li et al. (2015)
2015	USA	Gyrovirus 9	Gyrovirus homsa5 (GyH5)	*Homo sapiens*	Feces	Unknown	Phan et al. (2015)
2018	USA	Gyrovirus 10	Gyrovirus anas 1 (GyA1)	Anas *chauna torquata*	Plasma	Neural symptom	Goldberg et al. (2018)
2018	USA	Ashy storm petrel gyrovirus	Gyrovirus hydho1 (GyHy1)	Hydrobates homochroa	Cloacal swab	Unknown	Waits et al. (2018)
2019	Guiana	Gyrovirus 11	Gyrovirus myferr1 (GyM1)	Myrmoderus ferrugineus	Cloacal swab	Unknown	Truchado et al. (2019)
2020	Brazil	Pigeon gyrovirus	Gyrovirus pigeon 1(GyPi1)	Pigeons	Serum	Unknown	Loiko et al. (2020)
2020	Brazil	Gyrovirus 13	Gyrovirus anas 2 (GyA2)	Anas Pekin Ducks	Spleen	Unknown	Cibulski et al. (2020)
2022	Hungary	–	Gyrovirus phasi 1(GyPh1)	*Phasianus colchicus*	Mixed	Unknown	Fehér et al. (2022)
2023	New Zealand	–	Gyrovirus Mega 1(GyMe1)	*Megadyptes antipodes*	Mixed	Unknown	Wierenga et al. (2023)

## 4 Epidemiology

### 4.1 Prevalence and distribution

**Figure 3:**
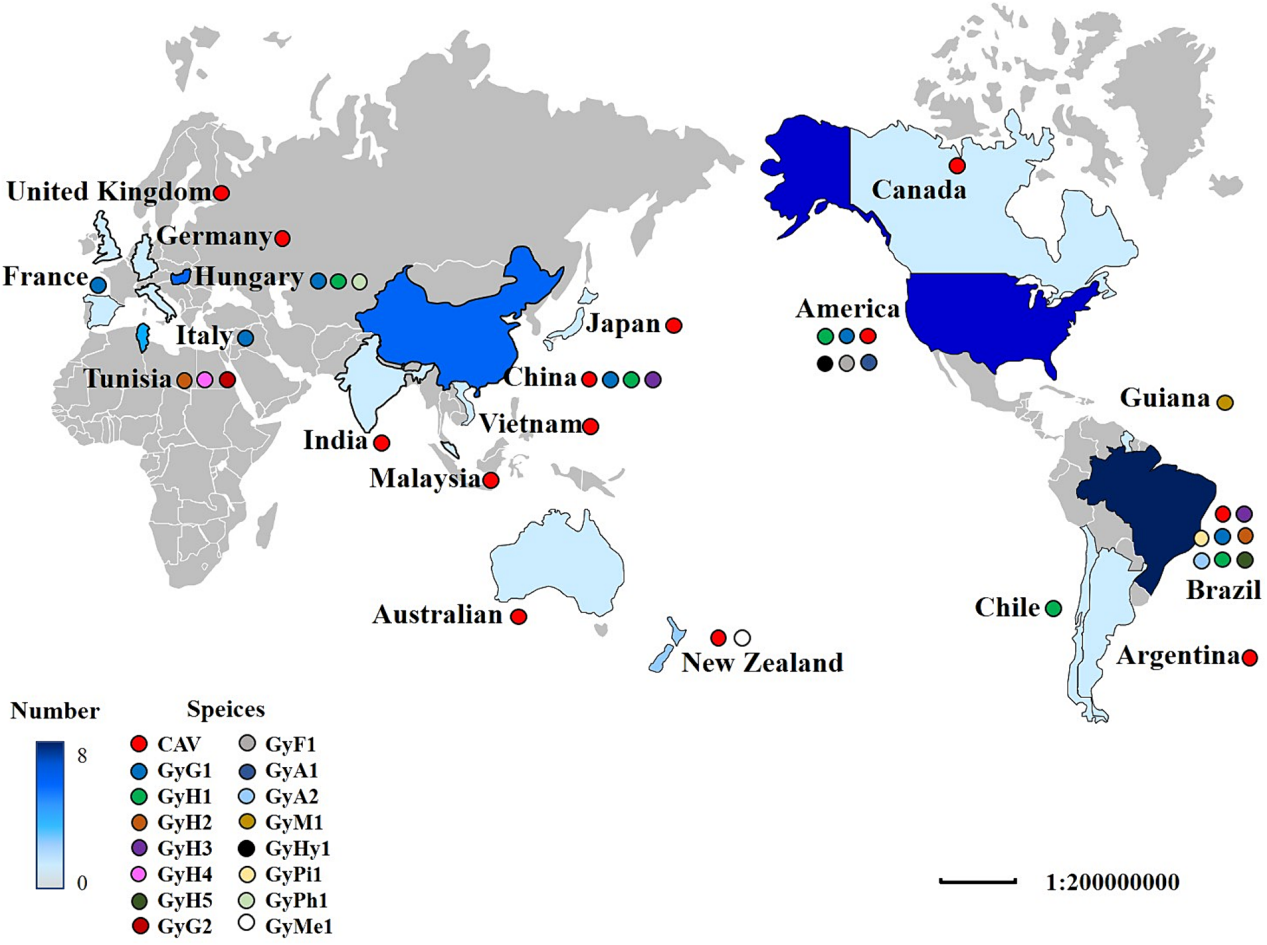
The distribution of GyV around the world. A colored circle represents the GyV of one species. The shade of blue indicates the number of GyV. The darker the color, the greater the number.

In the last decade, developing high-throughput sequencing (HTS) technology has greatly expanded our knowledge of the GyV that lives with and around us (Table 1) (Kumar et al., 2017). GyV has been documented in 19 countries, with Brazil having the highest number of GyV species (8), followed by the United States (6) and China (4) (Figure 3). The global distribution of GyV is supported by the fact that the USA, Brazil, and China are major poultry producers and exporters, as well as the intense borderless dissemination of commercial chicken breeds supports the idea that GyV is distributed worldwide.

The epidemiological investigation of GyV in the NCBI database only three viruses [CAV, GyG1, and GyH1 (Zhou et al., 1996; Ducatez et al., 2008; Eltahir et al., 2011; dos Santos et al., 2012; Ye et al., 2015; Yao et al., 2016, 2019; Ou et al., 2018; van Dong et al., 2019; Sreekala et al., 2020; Tan et al., 2020; Ji et al., 2022; Wang et al., 2022; Zhang S. et al., 2022; Zhang M. et al., 2022; Hien et al., 2023; Li et al., 2023; Sun et al., 2023; Xu et al., 2023; Yan et al., 2023; Zhang et al., 2023)]. The details are displayed in Table 2. Most reports of GyV have come from China, probably due to the detection and active search for the virus. Epidemiological investigations of GyV have not yet been reported by other countries, due to the lack of generally accepted evidence of pathogenicity and detection methods (e.g., detection primers), but this does not indicate that they are exempt of the virus. In fact, the existing surveys show that the rate of CAV infection remains high in some countries, notably China, Vietnam, and India. This is not surprising as there are no effective preventive measures against CAV in some countries to date (see the Section 7). The infection rate of GyG1 varies greatly in different regions, particularly in Brazil and New Zealand, recording significantly higher than in China. Although a link between GyG1 and related diseases has not yet been established, the high infection rate in poultry is a cause for concern.

**Table 2:** Epidemiological findings of GyVs (CAV, GyG1, and GyH1) over the last decade.

Sampling time	Country	Detection method	Positive rate		Disease	References
			Total	Co-infection		
**CAV**						
2011	China	PCR	10.2% (47/460)	–	Sick	Eltahir et al. (2011)
2014–2015	China	PCR	13.3% (96/722)	58.3% (65/96)	Sick	Yao et al. (2019)
2010–2015	China	PCR	52.5% (72/137)	–	Sick	Ou et al. (2018)
2016–2017	China	PCR	40.0% (91/227)	–	Sick	Tan et al. (2020)
2016–2017	India	PCR	60% (39/65)	–	Sick	Sreekala et al. (2020)
2016–2018	Vietnam	qPCR	47.5% (157/330)	–	Sick	van Dong et al. (2019)
2018–2019	China	PCR	12.2% (21/172)	38.1% (8/21)	Sick	Yan et al. (2023)
2017–2020	China	PCR	9.4% (41/437)	–	Sick	Wang et al. (2022)
2018–2020	China	PCR	17.1% (60/350)	36.7% (22/60)	Sick	Zhang M. et al. (2022)
2020–2021	China	qPCR	65.4% (375/573)	–	Sick	Sun et al. (2023)
2020–2021	Vietnam	PCR	42.5% (20/47)	–	Sick	Hien et al. (2023)
2020–2022	China	qPCR	13.4% (115/854)	40.8% (47/115)	Sick	Li et al. (2023)
2020–2022	China	PCR	62.7% (27/43)	44.4% (12/27)	Sick	Xu et al. (2023)
**GyG1**						
2012	Brazil	PCR	81.4% (127/156)	–	Health	dos Santos et al. (2012)
2012	Netherlands	PCR	42.9% (9/21)	–	Sick	dos Santos et al. (2012)
2015	China	PCR	18.5% (10/54)	–	Sick	Ye et al. (2015)
2015–2016	China	PCR	12.2% (55/448)	81.8% (45/55)	Sick	Yao et al. (2016)
2018–2019	China	PCR	9.8% (17/172)	23.5% (4/17)	Sick	Yan et al. (2023)
2022	China	PCR	12.6% (8/63)	–	Unknown	Ji et al. (2022)
**GyH1**						
2018–2019	China	PCR	3.4% (6/172)	33.3% (2/6)	Sick	Yan et al. (2023)
2017–2019	China	ELISA and qPCR	9.3% (203/2,192)	–	Sick	Zhang et al. (2023)
2022	China	ELISA (antibodies)	0.6–7.7% (12/2,055–158/2,055)	–	Sick	Zhang S. et al. (2022)

### 4.2 Co-infection

Epidemiological investigations have shown that usually GyV appear in coinfection with other pathogens (Table 2). Numerous studies have reported co-infection of CAV with various avian viruses, including low pathogenicity influenza virus (H9N2), infectious bronchitis virus (IBV), fowl adenovirus (FAdV), avian leukosis virus subgroup J (ALV-J), Marek’s disease virus (MDV), reticuloendotheliosis virus (REV), avian reovirus (ARV), infectious bursal disease virus (IBDV), and GyH1 (Gholami-Ahangaran, 2015; Erfan et al., 2019; Su et al., 2019a; Li et al., 2020; El-Azm et al., 2022; Yang et al., 2023). The enhancement of pathogenicity following avian dual infections with CAV and other pathogens appears to be synergistic, and CAV lesions exhibit greater severity due to the effects of the co-infecting virus (Erfan et al., 2019; Zhang et al., 2021; Yang et al., 2023). Co-infected chicks with CAV and ARV exhibited severe reductions in packed cell volume and tissue damage (McNeilly et al., 1995). MDV and CAV co-infection exert a negative impact on the infectivity and pathology of avian respiratory viruses (Shulman and Davidson, 2017). CAV and ALV-J co-infection significantly inhibited humoral immunity (Zhang et al., 2021). CAV and GyH1 co-infection induced a more severe impact on the development of immune organs and the pathogenicity of multiple chicken organs (Yang et al., 2023). These co-infections may lead to an exacerbation of a range of clinical manifestations, thereby accelerating the severity of the disease and increasing morbidity and mortality in affected chickens.

A possible hypothesis is that CAV inhibits host cytokines involved in antiviral response and those involved in IFN synthesis (see the section 5), thereby promoting the proliferation of other viruses, but the specific mechanism needs further exploration. The mechanism of other GyV co-infection with other agents is currently unknown. One alternative hypothesis is that GyV is an opportunistic virus infecting or growing during the infection by the other pathogen or under conditions enabling the other pathogen such as compromised immune response. Even if they have no obvious pathogenic characteristics, more research is needed to understand the mechanisms of co-infection of GyV so that their hazards can be further assessed.

### 4.3 Host range and transmission route

While there is currently unclear evidence on whether these viruses replicate in humans, the potential for zoonotic transmission of these viruses and their impact on public health safety cannot be ignored. GyV is widely distributed worldwide and has an apparent pan-tropism at the host level. Although chickens are the main natural hosts of CAV, other birds, such as turkeys and quails, may also be infected by this virus (Gholami-Ahangaran, 2015). Increasing epidemiological evidence suggests that GyG1 and GyH1 can also infect chickens (Ye et al., 2015; Yao et al., 2016; Li et al., 2021; Yan et al., 2023). Chickens may be an ideal model for studying GyV transmission, virulence, immunity, and pathogenesis. Furthermore, GyV has been found in various birds, such as pigeons, ducks, fulmars, ferruginous-backed antbird, ashy storm-petrels, pheasant and penguin, suggesting that they appear to be uniquely infectious to birds (Li et al., 2015; Lima et al., 2017; Goldberg et al., 2018; Waits et al., 2018; Truchado et al., 2019; Cibulski et al., 2020; Loiko et al., 2020). Several species of GyVs, including CAV, GyG1, GyH1, GyH2, GyH3, GyH4, and GyH5, have been detected in human diarrheic feces (Rijsewijk et al., 2011; Chu et al., 2012; Phan et al., 2012, 2015; Gia Phan et al., 2013). One hypothesis suggests that the viral genomes in these feces originates from chicken ingestion. Evidence in support of this theory is the fact that GyV has also been found in chicken meat and the feces of other chicken-consuming animals such as snow ferrets, snakes, dogs, and cats (Fehér et al., 2014, 2015; Fang et al., 2017; Wu et al., 2019). However, a study has shown that GyH1 can infect chickens and mice, causing gastroenteritis, demonstrating its ability to infect across species (Yuan et al., 2021).

The widespread prevalence of GyV and its detection in various biological samples may be due to a variety of transmission routes. CAV can be transmitted either vertically from laying hens or horizontally in chicks with no maternal antibodies (Schat, 2009). Previous studies have displayed that experimentally infected males transmit CAV to their offspring through semen until they produce CAV antibodies between 8 and 14 dpi (Cardona C. J. et al., 2000). Bird reproductive tissues may contain the virus in the absence of seroconversion and the antibodies against CAV do not prevent vertical transmission (McNulty, 1991; Cardona C. et al., 2000; Brentano et al., 2005). It has been proposed that a well-adapted relationship between the host and pathogen exists when conditions of low-stress, low challenge dose of virus and flocks with chronic infection are met. Under these conditions, CAV may evade immune system detection until hormonal activity, associated with the onset of laying, possibly allows the reactivation of virus replication, thus permitting virus transmission to the progeny (Miller and Schat, 2004).

The primary route of horizontally transmitting CAV is through fecal-oral transmission. The detection of GyV in various animal feces and in the mouth and anus of wild birds suggests that other GyVs may also be transmitted in this manner (Phan et al., 2012; Fang et al., 2017; Lima et al., 2017; Niu et al., 2019; Truchado et al., 2019; Wu et al., 2019). CAV can be spread through feather dust and dander in addition to the oral-fecal and vertical transmission routes (Davidson et al., 2008; Davidson, 2009). According to the indication, CAV is present in the feather follicle tissues and spreads in an infectious form, causing tissue damage similar to that caused by MDV (Davidson, 2009). CAV and GyG1 often contaminate commercially available poultry vaccines (Marin et al., 2013; Varela et al., 2014; Su et al., 2019a); the use of vaccines contaminated with the virus is one of the important transmission routes of GyV to spread around the world.

### 4.4 Serotype

The majority of GyV isolates have only one serotype. Spackman, E. et al. reported the second serotype of CAV based on its physicochemical characteristics and pathology (Spackman et al., 2002). However, based on the lack of antibody detection in the enzyme-linked immunosorbent assay (ELISA), negative PCR results, and the lack of information on the genome, it is not certain that this pathogen is a CAV. The single serotype suggests that elicits antibodies against this single vaccine may eradicate all GyVs. However, CAV is maintained and possibly replicated in MDCC-MSB1 (MSB1) cells cultured in the presence of neutralizing antibody (NA) (Van Dong et al., 2020). Anti-CAV antibodies do not prevent vertical transmission and prevalence of CAV, but are protective against CAV-induced disease (Ingberman et al., 2021). In addition, antibody test could not detect the presence of ongoing CAV infections. Cardona et al. reported that ovaries of antibody-positive and -negative SPF hens can be positive for virus DNA. This finding in combination with observations from the SPF poultry industry that birds often seroconvert when in production.

### 4.5 Genetic evolution

**Figure 4:**
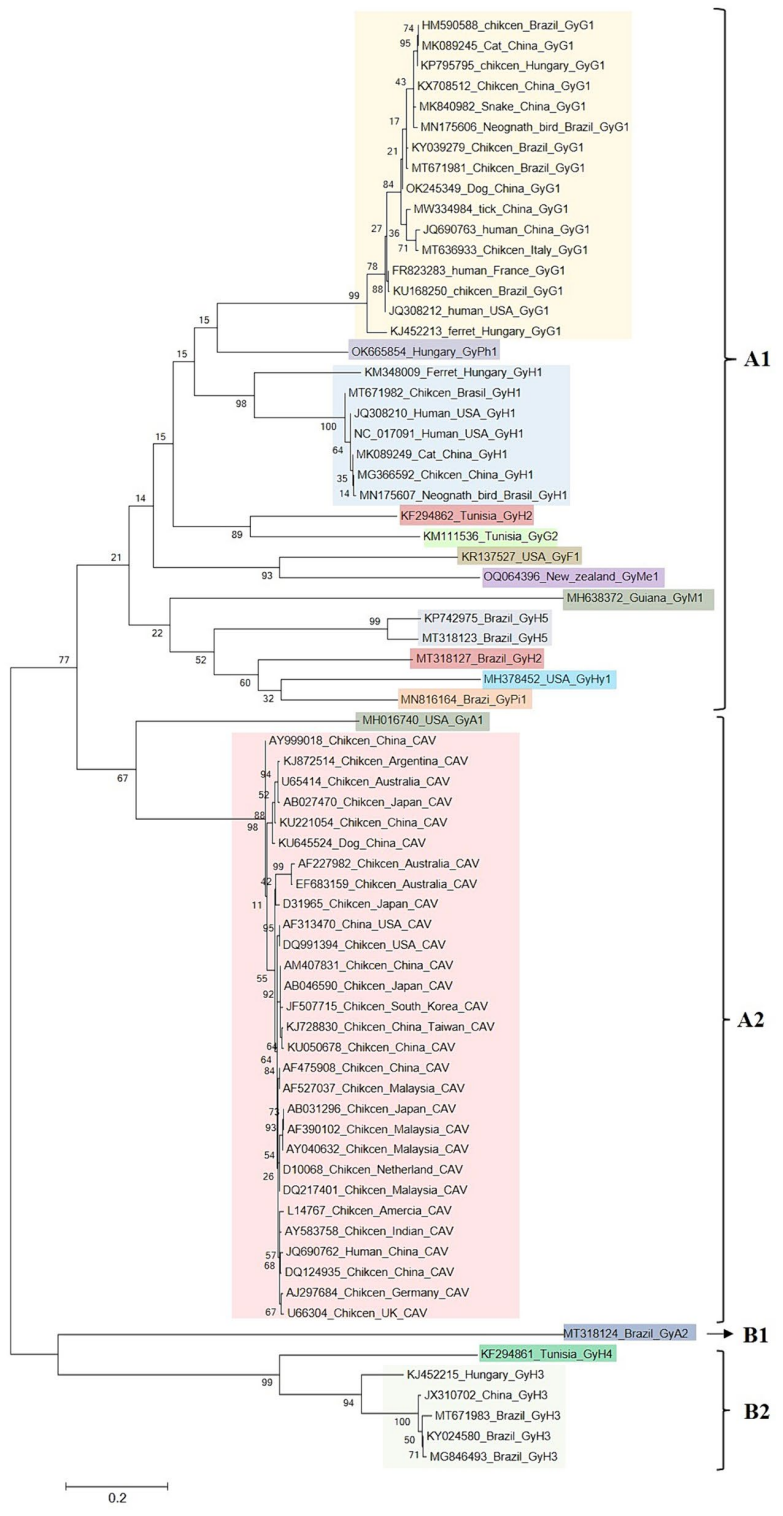
Maximum likelihood phylogenetic analyses of GyV. Duplicate sequences obtained with the same date and location were removed using BioAider with a 99% threshold. The final 71 reference sequences of GyV were collected for data analysis. All GyV sequences (3,401 bp) were aligned using Clustal W in MEGA 6. The trees were constructed with MEGA 6, using the maximum likelihood GTR + G4 as a substitution model for the genome. Phylogenetic trees Bootstrap values (with a basis on 1,000 replicates) superior to 60% are shown.

To enhance comprehension of the genetic evolution of GyV, a phylogenetic tree was constructed by screening 71 representative strains (Duplicate sequences obtained with the same date and location were removed using BioAider with a 99% threshold) from the NCBI database to construct a phylogenetic tree. The maximum likelihood phylogenetic tree of the GyV could be divided into two major clades, displaying some host-specific demarcation but lacking any regional demarcation (Figure 4). With the exception of GyA2 in clade B, all GyV strains found in birds were clustered in clade A. It is possible that these viruses were initially transmitted among various bird species and could adapt to various avian hosts. Among the GyV found in human feces, GyH1, GyH2, and GyH5 were in clade A and GyH3 and GyH4 were in clade B. GyH1 has been shown to infect chickens and should therefore be considered an avian pathogen, although it was originally found in human feces. In addition, some GyVs nucleotide sequences of the different host isolates were highly homologous (Table 3), and these strains were closely related from the phylogenetic tree (Figure 4), suggesting their zoonotic potential.

**Table 3:** The homology of GyV comparison of human and other animals.

Virus	GenBank (animals)	Homology % (comparison with human)	
CAV			
		JQ690762 (human)	
	AY583758 (chicken)	98.5	
	KU645524 (dog)	96.3	
GyG1			
		FR823283 (human)	JQ308212 (human)
	MT627326 (chicken)	99.3	99.5
	KU168250 (chicken)	99.2	97.8
	OK245349 (dog)	98.2	96.7
	MW334984 (tick)	96	95.9
	MK840982 (snake)	97.1	95.7
	MK089245 (cat)	95.7	94.2
	KJ452214 (ferret)	95.1	93.7
GyH1			
		JQ308210 (human)	
	MK089249 (cat)	99.5	
	MG366592 (chicken)	98.4	
	MT671982 (chicken)	98.4	

Nucleotide alignments and homology analysis were performed with MegAlign.

The GyV family appears to be ancient, and millions of years of evolution have led to its current genetic diversity. An important factor in the high diversity of the virus is its high mutation rate or frequent recombination between different strains. Like other ssDNA virus, GyV has a high rate of evolution (CAV, 6.09 × 10^−4^ substitutions/site/year, and GyG1, 2.784 × 10^−4^ substitutions/site/year), which is closer to closer to those of RNA viruses than double stranded DNA viruses (Bédarida et al., 2017; Techera et al., 2021; Yan et al., 2023). Previous studies have shown evidence of genetic recombination events in GyV, some of which have been observed in dogs, ferrets, and humans (Li et al., 2017; van Dong et al., 2019; Tan et al., 2020; Liu et al., 2022; Shah et al., 2023; Yan et al., 2023). Moreover, it has been reported that the genomes of CAV and GyG1 contain similar recombination regions, that include coding regions at the tail of the VP1 protein, non-coding regions (UTR), and overlapped coding regions (Yan et al., 2023). The potential gene exchange regions contains RCR, DR and promoter/enhancer, which are associated with viral replication and transcription. Conflicts between transcription and replication enzyme complexes or interruptions in replication caused by single-strand breaks in the replicative form dsDNA template strand can lead to premature detachment of replication complexes. If replication is restarted after these complexes reattach to a template molecule other than the one that initially started replication, the resulting fully replicating genome will be a recombinant produced by a mechanism known as copy-choice (Martin et al., 2011). In addition, these viruses may have similar evolutionary mechanisms, which means that recombination may also occur between different GyVs.

## 5 Immunopathogenesis

### 5.1 Innate immunity

**Figure 5:**
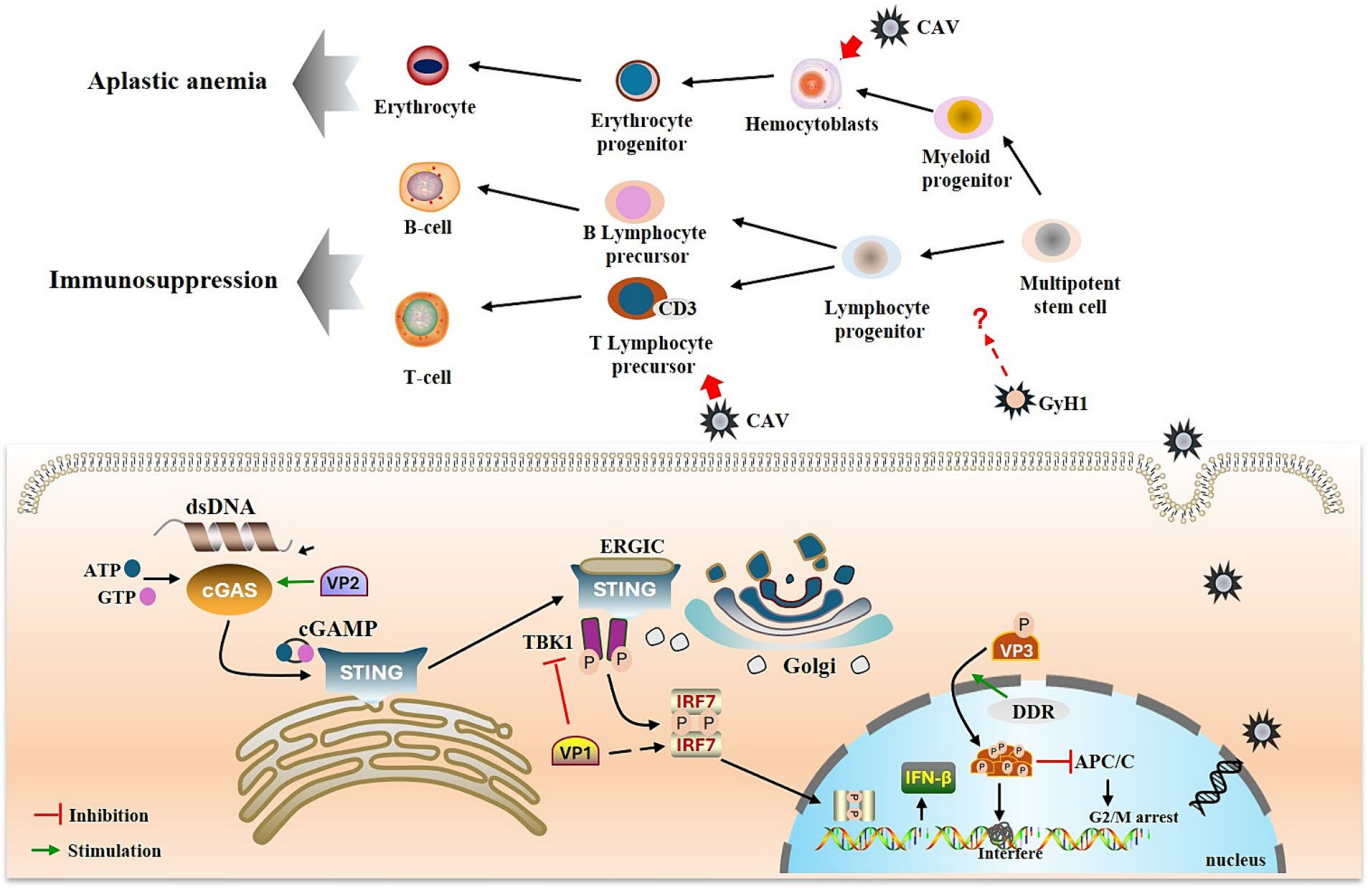
Immunopathogenesis of GyV. GyV genome enters the nucleus for replication, forming dsDNA, which activates the cGAS-STING pathway. The viral protein VP2 promotes activation of the cGAS-STING pathway and thus IFN-β production. VP1 inhibits IFN-β production by inhibiting TBK1 phosphorylation and interacting with IRF7. DDR and T108 phosphorylation promote nuclear localization of apoptin and activated apoptin starts to accumulate within the nucleus. This accumulation leads to the formation of apoptin multimers, which interfere with DNA transcription, synthesis and repair, thereby inducing cell death. Additionally, phosphorylated apoptin inhibits the APC/C and prevents the continuation of the cell cycle. The green arrow represents promotion, and the red horizontal line represents inhibition.

Innate immunity is the first line of defense against pathogenic microorganisms. CAV infection interferes with the innate immune response of the host. CAV has been reported to negatively affect vaccine-induced innate and acquired immune responses (Zhang et al., 2017). Compared with other immunosuppressive viruses such as IBDV and MDV, the expression of IFN-α, IFN-β, and other inflammatory cytokines (IL-1β and IL-6) increase gradually in chickens on day four after CAV infection but are significantly lower on days seven and eleven post-infection, suggesting that CAV negatively affects the innate immunity of the host (Giotis et al., 2015). Additionally, CAV blocks the induction of IFN-I and ISGs at 72 h post infection in MSB1 cells (Giotis et al., 2018) (Figure 5).

Innate immunity deploys various pattern recognition receptors to detect extracellular or intracellular pathogens. Cyclic GMP-AMP synthase (cGAS), a key DNA sensor, detects cytosolic viral DNA and activates the adaptor protein stimulator of interferon genes (STING) to initiate interferon (IFN) production and innate antiviral responses of the host (Chen et al., 2016). Recently, it was shown that CAV VP1 inhibits the cGAS-STING pathway, which mediates the activation of downstream antiviral genes by interacting with IRF7 and weakening the innate antiviral response (Liu et al., 2023a). Another study demonstrated that CAV VP1 antagonizes IFN production by inhibiting TBK1 phosphorylation (Chen et al., 2023). However, the regulation of STING by viral proteins seems contradictory (Cheng et al., 2020). The nonstructural protein VP2 of CAV can upregulate the expression of IFN-β through its interaction with cGAS (Liu et al., 2023b). This opposite regulatory effect is also observed in other DNA viruses. For example, while HSV-1 UL46 has been suggested to negatively regulate STING protein levels, infected cell protein 0 (ICP0), ICP4 and US3 protein kinase (US3-PK) encoded by HSV-1 have been reported to stabilize STING (Kalamvoki and Roizman, 2014; Deschamps and Kalamvoki, 2017). The PCV2 infection can activate the cGAS/STING signaling pathway to promote IFN-β production (Huang et al., 2018), whereas recent studies have shown that PCV2 CAP protein can inhibit cGAS phosphorylation by activating the PI3K/AKT signaling to promote other DNA virus infections (Wang et al., 2021).

### 5.2 Adaptive immunity

The adaptive immune system has two main mechanisms cellular and humoral immunity, mediated by T and B cells, respectively (Bonilla and Oettgen, 2010). The thymus is the primary site of T cell differentiation and maturation, and is also one of the target tissues of CAV. The T lymphocyte progenitor cells in the thymus are particularly susceptible to CAV infection; cells expressing cytoplasmic CD3 (CD3 cp) are among the earliest cells in which the CAV antigen is detected (Taniguchi et al., 1983; Jeurissen et al., 1989). Lymphocyte depletion in the thymic cortex is a characteristic lesion caused by CAV infection; GyH1 infection also causes this lesion (Pope, 1991; Li et al., 2021). The destruction of lymphocytes may be related to apoptosis induced by VP3 (apoptin). Apoptotic corpuscles were observed in the thymus of CAV-infected chickens by electron microscopy, and similar changes were also observed in CAV-infected lymphoblastoid cell lines (Jeurissen et al., 1992). Furthermore, transfection of cells using an expression vector containing only the VP3 gene resulted in transient expression of the VP3 protein and apoptosis (Noteborn et al., 1994). The mechanism of apoptosis induced by apoptin appears to linked to the DNA damage response (DDR). It has been reported that DDR promotes the nuclear localization of apoptin and subsequently phosphorylates apoptin via a phosphokinase, thereby facilitating its accumulation in the nucleus (Kucharski et al., 2011, 2016). This accumulation leads to spontaneous irreversible semi-random aggregation of approximately 20–40 apoptin subunits to form a multimer containing eight independent non-specific DNA binding sites, which preferentially bind strand ends and interfere with DNA transcription, synthesis, and repair to induce cell death (Leliveld et al., 2003a,b; Guelen et al., 2004). Furthermore, targeting of the anaphase promoting complex/cyclosome (APC/C) by apoptin to promyelocytic leukemia protein (PML) nuclear bodies suggests modification of the mitotic functions of the APC/C and inhibition of APC/C activity, which could serve to facilitate apoptotic programming (Heilman et al., 2006).

In contrast to the significant reduction in T cell numbers following infection, there was no substantial reduction in B cell numbers. Neutralizing antibodies were first detected 3 weeks after inoculation of CAV into one-day-old chickens (Yuasa et al., 1983). The appearance of CAV antibodies in the serum coincided with the disappearance of the virus from the blood and from several tissues, although infectivity was observed to persist in some organs for some time (Yuasa et al., 1983). Besides, maternal antibodies have been demonstrated to provide complete protection against CAV-induced disease (Pope, 1991). The B cells and their precursors are less susceptible to CAV infection, a factor critical for the survival and ultimate recovery of infected birds. However, unlike CAV, which selectively destroys T lymphoid precursor cells, GyH1 appears to affect all lymphocytes. GyH1 infection has been linked to immunosuppression, which is characterized by severe depletion of lymphocytes in the thymic cortex, lymphoid areas in the spleen, and depletion in the bursa of Fabricius as well as bone marrow failure (Li et al., 2021). GyH1 and CAV may affect immune and hematopoietic function by mediating the proliferation and differentiation of stem and progenitor cells in bone marrow. Therefore, the bone marrow is an important target for future studies regarding pathogenesis.

## 6 Pathogenicity and potential risks

Similarities between animal and human sequences have suggested their zoonotic potential, but without actual reports of diseases associated with GyVs. CAV is an immunosuppressive virus that causes bone marrow hypoplasia and systemic lymphocyte atrophy in chickens, resulting in anemia, immunosuppression, and an approximately 10% increase in mortality (Pope, 1991). These have been described in detail in previous studies. Recently, several studies have reported links between GyV and various diseases, primarily affecting the nervous, digestive, and immune systems (Table 1). In these studies, GyG1, GyF1, and GyA1 were found to be associated with neurological symptoms, including ataxia, paralysis, and head and neck tilt (Abolnik and Wandrag, 2014; Li et al., 2015; Goldberg et al., 2018). The genomes of CAV, GyG1, and GyH1 have shown high abundance in chickens with transmission viral proventriculitis (TVP), indicating their potential role in TVP (Yan et al., 2022). Several epidemiological investigations have identified that GyG1 and GyH1 in chickens with clinical manifestations of depression, wasting, and stunted growth (Table 2).

CAV and GyH1 are immunosuppressive in chickens (Balamurugan and Kataria, 2006; Li et al., 2021; Yuan et al., 2021). Immunosuppressive viruses usually increase secondary or opportunistic viral, bacterial, or fungal infections; they can also cause vaccination failures (Goryo et al., 1987; McIlroy et al., 1992). The use of CAV-contaminated NDV attenuated vaccine resulted in caused marked signs of chicken infectious anemia (CIA) in chickens and reduced NDV antibody titers (Su et al., 2019b). Additionally, various GyVs (CAV, GyG1, GyG2, GyH1, and GyH3) have been identified from chicken meat in supermarkets by metagenomics (Zhang et al., 2014). According to report that CAV were detected as contaminants in 6/32 live vaccines and in 1/3 inactivated vaccines. The CAV genome loads ranged from 6.4 to 173.4 per 50 ng of vaccine DNA (equivalent to 0.07 to 0.69 genome copies per dose of vaccine). Likewise, AGV2 genomes were detected in 9/32 live vaccines, with viral loads ranging from 93 to 156,187 per 50 ng of vaccine DNA (equivalent to 0.28–9,176 genome copies per dose of vaccine) (Varela et al., 2014). These findings indicate the potential risk of GyVs to food safety and the quality of biological products. Although most GyVs have not yet been characterized as harmful to humans or other economically beneficial animals, it is necessary to give more attention and resources due to the possible risk of GyV in poultry and zoonotic potential in humans.

## 7 Culture, detection, and prevention

The pathogenicity of most GyV infections remains unknown due to several limitations, including the scarcity of viral isolates and the lack of viral detection and culture systems. These limitations make it difficult to obtain the appropriate tools, animals, and conditions for experimental infections. In fact, only CAV can be cultured on MDCC-MSB1 (Renshaw et al., 1996), and no other GyV has been successfully cultured *in vitro*. In a recent study, eight cell lines were selected for GyH1 culture. However, GyH1 did not replicate for more than three generations in any of them, indicating that a reliable cell culture system for GyH1 culture remains an unachieved goal (Yuan et al., 2021). The availability of infectious clones that can be used to generate recombinant virus cultures is critical for preparing pure cultures of GyVs so that they can be studied in the absence of contaminants that may could be present in natural isolates. Several infectious cloning experiments of ssDNA viruses have successfully achieved viral rescue *in vivo* and *in vitro*, such as CAV, PCV, and Torque Teno Sus Virus (TTSuV) (Noteborn et al., 1991; Fenaux et al., 2003; Huang et al., 2012; Jiang et al., 2019). Constructing infectious clones in the absence of viral culture cell lines is one of the most effective ways to explore the pathogenesis of these newly discovered viruses.

Virus isolation in cell culture or embryonating eggs is considered the gold standard procedure for virus detection and has been routinely used; however, it is cumbersome, expensive, and time-consuming (Goryo et al., 1985; McNulty et al., 1989; Rosenberger and Cloud, 1989). Numerous methods for detecting CAV infection have been reported over the past two decades, including immunofluorescence, *in situ* hybridization, ELISA, sensitive loop-mediated isothermal amplification (LAMP), and gold immunochromatography; however, specificity, sensitivity, and cost may limit the use of these methods (Sander et al., 1997; Vagnozzi et al., 2018; Han et al., 2019; Wu et al., 2022; Angsujinda et al., 2024). Early and sensitive detection of the virus is an important parameter to control the outbreak of disease in chickens. Among the latest CAV detection technologies, nanoparticle assisted PCR (nano-PCR) and droplet digital PCR (ddPCR) have been shown to be more sensitive than traditional methods and may be valuable in detecting CAV contamination in vaccines (Li et al., 2019; Luan et al., 2022). Currently, detection methods for other GyVs are limited. Polymerase chain reaction (PCR) is usually the detection method used in several epidemiological investigations of GyV (Yao et al., 2016; Tan et al., 2020; Zhang M. et al., 2022; Yan et al., 2023). Additionally, the development of combined multiple virus detection technology is of great significance for immunosuppression of CAV and GyH1-induced multi-pathogen mixed infection. Although multiplex PCR (mPCR) has been successfully established for the detection of FADV, ARV, IBDV, and CAV, there are currently no combined detection methods available for multiple GyVs (Caterina et al., 2004; Cong et al., 2018; Luan et al., 2022). It is important to note that the current identified GyVs share the synteny of their genomes, and more attention should be paid to mixed infections involving multiple GyVs.

Vaccination is a safe and effective control measure used to curtail the severity of immunosuppressive symptoms caused by GyV in young chicken. Attenuated live and inactivated vaccines against CAV disease have shown protection against vertical transmission of the virus (McNulty, 1991; Noteborn et al., 1998; Zhang et al., 2015). However, these vaccines have some limitations that constitute a challenge to vaccine development and widespread application, including the virulence reversion of the virus, the inability of CAV strains to grow to high titer levels in embryonic or cellular cultures, and the timing of vaccination in young birds that are positive for maternal antibodies (Hussein et al., 2003; Vaziry et al., 2011; Sawant et al., 2015). To circumvent these limitations, different experimental studies have reported the efficacy of subunit, nucleic acid, and recombinant vaccines in inducing high specific CAV antibody titers in vaccinated chickens (Lacorte et al., 2007; Lee et al., 2011; Moeini et al., 2011a,b; Tseng et al., 2019). However, these vaccines have not yet been formally approved for large-scale production.

Currently, there is no specific treatment or vaccine available for other GyVs. Although there is evidence that the GyH1 virus is pathogenic to poultry, the pathogenicity of most GyV remains unclear. However, it cannot be ignored that they have been prevalent in chickens for a long time and may play a potential role in some diseases. Therefore, it is necessary to develop additional assays to obtain more epidemiological data that will clarify the clinical significance of these viruses and to inform effective prevention and control strategies. Here, we provided GenBank numbers for representative GyVs sequences and designed primers for PCR detection using primer 6.0 software to aid in the ongoing focus on these viruses (Table 4). Moreover, prior to the availability of vaccines and effective treatment drugs, strategies should be devised to prevent and control GyV infection focusing on strengthening farm management and implementing strict quarantine measures for chickens, including proper hygiene, sanitation and disinfection, rigorous biosecurity planning and regular surveillance of viral infections, followed by judicious use of antiviral drugs.

**Table 4:** Primers for detection of GyV.

Virus	GenBank (size)	Primer sequence (5′→3′)	Position (size)	Source
CAV	NC_001427 (2,319 bp)	F: GACTGTAAGATGGCAAGACGAGCTC R: GGCTGAAGGATCCCTCATTC	823–1,498 (676 bp)	References
GyG1	HM590588 (2,383 bp)	F:CGTGTCCGCCAGCAGAAAC R:GGTAGAAGCCAAAGCGTCCAC	656–1,001 (346 bp)	References
GyH1	NC_017091 (2,359 bp)	F:GACACAGACTGCGACGAAGA R:ATGCTCCTGGCTGTCTAGAT	968–1,399 (432 bp)	References
GyH3	JX310702 (2034 bp)	F: GTGGTATCGAAGTGGAAAGTACC R: CCCCCTGATACATACTGTACATA	1,061–1,345 (285 bp)	References
GyH2	KF294862 (2,282 bp)	F: TCCATCATTCTCAGCGTTAT R: CGTAGATTGTGGCTATTGTG	1,875–2,092 (218 bp)	Design
GyH4	KF294861 (2020 bp)	F:CAAGATTCGTATTGGCAAGT R:CATTCACTCCGCACCTTA	1,580–1,953 (374 bp)	Design
GyG2	KM111536 (2,439 bp)	F:CGAAGTGTAAGCCGAAGG R:CGTTGTTCCACCAGTTGA	2,035–2,374 (340 bp)	Design
GyF1	KR137527 (2,218 bp)	F:TTGACTGGTGGAGATGGT R:CCTGTGATTGTGCTGGTT	1,276–1,606 (331 bp)	Design
GyH5	MT318123 (2,215 bp)	F:CTGCCTTATACTGGTGGAAT R:TTGGTAGTTCTTGCTGGTAA	1,281–1,761 (481 bp)	Design
GyA1	MH016740 (2,195 bp)	F:CCTCATTCGGAACACTCTC R:TGGTTGGTCTTGGTCTGT	1,745–2,016 (272 bp)	Design
GyHy1	MH378452 (2,394 bp)	F:CCATCCTTCGCAACACTAT R:CACCTTGACTGTATCTTAACTG	1,569–1,900 (332 bp)	Design
GyM1	MH638372 (1873 bp)	F:ACCACCGAAGACGATACT R:TAGGAGGTTGCCGAATGA	750–1,006 (257 bp)	Design
GyPi1	MN816164 (2,573 bp)	F:TAAGAGCAGCGAGCATTG R:TGGTAGCGGATACATTAGTG	1,414–1,691 (278 bp)	Design
GyA2	MT318125 (2,195 bp)	F: AGACATAGACGACGATTCC R: AGTTCATTACTGCTCTCCAT	615–912 (298 bp)	Design
GyPh1	OK665854 (2,353 bp)	F:ATACGAATGGTGGAGATGG R:TAGTGTGGCGAATGATGG	1,431–1,908 (478 bp)	Design
GyMe1	OQ064396 (2,573 bp)	F:TAAGAGCAGCGAGCATTG R:TGGTAGCGGATACATTAGTG	1,414–1,691 (278 bp)	Design

Designed primers for GyV detection using primer 6.0 software.

## 8 Conclusion

Since the identification of the first member (CAV) of the genus GyV, they have been detected in a variety of biological samples, showing their global distribution and host diversity. They are particularly highly prevalent in chickens, and co-infection of GyV with other pathogens is also common in chickens. Therefore, we should focus on monitoring the prevalence and co-infection of GyV with other pathogens, as well as continue to closely monitor dynamic changes in genetic diversity and molecular epidemiology of dominant GyV strains. Meanwhile, as the exact pathogenesis of GyV remains to be elucidated, virus isolation of GyV from clinical samples or rescue of GyV using infectious clones should provide more insight to elucidate GyV pathogenesis and development of effective prevention and control techniques.
